# Root Starch Reserves Are Necessary for Vigorous Re-Growth following Cutting Back in *Lotus japonicus*

**DOI:** 10.1371/journal.pone.0087333

**Published:** 2014-01-31

**Authors:** Cécile Vriet, Alison M. Smith, Trevor L. Wang

**Affiliations:** Department of Metabolic Biology, John Innes Centre, Norwich, Norfolk, United Kingdom; University of Nottingham, United Kingdom

## Abstract

Perenniality and vegetative re-growth vigour represent key agronomic traits in forage legume (Fabaceae) species. The known determinants of perenniality include the conservation of the vegetative meristem during and after the flowering phase, and the separation of flowering from senescence. The ability of the plants to store nutrient resources in perennial organs and remobilize them may also play an important role in the perennial growth habit, and in determining the capacity of the plant to re-grow following grazing or from one season to the next. To examine the importance of stored starch, we examined the vegetative re-growth vigour following cutting back of a unique collection of *Lotus japonicus* mutants impaired in their ability to synthesize or degrade starch. Our results establish that starch stored in the roots is important for re-growth vigour in *Lotus japonicus.* We extended this analysis to a collection of *Lotus* (trefoil) species and two ecotypes of *Lotus japonicus* displaying a large variation in their carbohydrate resource allocation. There was a positive correlation between root starch content and re-growth vigour in these natural variants, and a good general correlation between high re-growth vigour and the perennial life-form. We discuss the relationship between perenniality and the availability of root carbohydrates for re-growth.

## Introduction

Perenniality and the ability to re-grow following grazing are traits of agronomic value in legumes (Fabaceae), a family that includes some of the most agriculturally important forage crops such as alfalfa (*Medicago*), clover, and trefoil (*Lotus*). It is likely that the ability of plants to store and remobilize carbon and nitrogen reserves in persistent organs plays an important role in determining their perennial lifestyle and/or their capacity for vigorous re-growth following grazing, cutting back, or winter die-back. Direct genetic and quantitative evidence about this characteristic is lacking. In the perennial forage legume alfalfa there are positive correlations between non-structural carbohydrate contents of roots in winter and re-growth vigour in spring, and the onset of shoot re-growth coincides with loss of non-structural carbohydrates from roots [1]–[4]. These observations have led to the suggestion that root carbohydrates represent an important carbon supply for re-growth. However, re-growth vigour in alfalfa also correlates with storage and remobilisation of nitrogen reserves in roots. It is not known whether carbon or nitrogen reserves are the more important for re-growth [5]–[7].

In order to determine the importance of carbohydrate reserves for re-growth vigour, we have made use of extensive genetic resources available in the genus *Lotus:* we have mutant lines of *Lotus japonicus* which differ profoundly in their capacity to synthesise or degrade starch [8] and we have assembled a collection of *Lotus* annual and perennial herbaceous species with similar growth habits. Cutting-back experiments on the *L. japonicus* starch mutants, in which the shoots of mature plants were removed to near ground level, revealed the importance of starch reserves for re-growth vigour. In addition, cutting-back experiments on the collection of *Lotus* species showed a positive correlation between root starch content and re-growth vigour. We suggest that root starch may be very important for maintenance of plant functions following defoliation, prior to the development of new photosynthetic capacity. Although the capacity to store starch is unlikely to be a primary determinant of the perennial habit, it may well confer adaptive advantages on perennial herbaceous plants.

## Materials and Methods

### *Lotus* Accessions and Species

Mutants of *L. japonicus* impaired in starch synthesis or degradation used in this study were previously described in Vriet *et al.* [8]. The starch synthesis mutants analyzed were *pgm1*, *pgi1*, *aps1* and *apl1*, which are deficient in plastidial phosphoglucomutase, plastidial phosphoglucoisomerase and the small and the large subunits of ADPglucose pyrophosphorylase (AGPase) respectively. The starch degradation mutants were *gwd1* and *gwd3*, which are deficient in glucan, water dikinase 1 and glucan water, dikinase 3 (also called phosphoglucan, water dikinase), respectively. The mutant alleles *pgm1-4, pgm1-5* and *aps1-1* almost eliminate starch from all organs, whereas the mutant allele *pgi1-1* strongly reduces starch content in leaves only. The *apl1-1* mutant has an 80% reduction in leaf starch content. The mutant alleles *gwd1-1 and gwd3-1* lead to a starch excess phenotype in leaves.

With the exception of *aps1-1*, analysis was performed on mutant and wild-type segregants from F2 populations, obtained by crossing the originally-selected mutants in the Gifu background [8] with the wild-type ecotype *L. japonicus* MG-20. For the *aps1* mutant, re-growth was compared with that of *L. japonicus* Gifu and MG-20 wild-type plants. All genotypes were at the second backcross stage except for *pgm1-4,* which was at the third backcrossing stage. The genotypes of individual plants were identified by sequencing or restriction analysis as described in Vriet *et al.* [8].

Seeds of the annual and perennial species of *Lotus* were from the Germplasm Resource Information Network (GRIN; http://www.ars-grin.gov/) of the United States Department of Agriculture/Agriculture Research Service (USDA/ARS), with the exception of seeds of *Lotus burttii* and *L. japonicus* which were from the John Innes Germplasm Resources Unit. Two ecotypes of *L. japonicus* (Regel) K. Larsen, Gifu (B129) and Miyakojima (MG-20), were used.

### Cutting-back Experiments and Biomass Measurements

Unless otherwise indicated, cutting-back experiments on mutants were carried out on 4- to 5-month old plants grown in a glasshouse in NL7 pots (Berrycroft Stores Ltd, Cambridge, UK) of F2 compost (Levington, Scotts Professional, Ipswich, UK) that were flowering and setting seeds. For each genotype, four to six plants were cut back at one to two cm above the stem base, and all the photosynthetic tissue removed. Prior to cutting and after six weeks of re-growth, the shoot fresh weight was measured and the starch content revealed by iodine staining. For the *Lotus* species, shoots of three-month-old plants were cut back. After 15 days of re-growth, plants were harvested and shoot fresh weights measured. The root systems were stained with iodine before and after cutting back. The collection was analysed in two batches, both of which contained annual and perennial species (Table 1). Two ecotypes of *L. japonicus* (MG-20 and Gifu) were grown in both batches. As a measure of “re-growth vigour”, shoot fresh weight after re-growth was expressed as a percentage of shoot weight at the time of cutting back.

**Table 1:** Re-growth after cutting back of a collection of *Lotus* species.

Species and experiment	USDA GRIN identifier	USDA GRIN or published life form	Initial shoot FW (g)	Shoot FW after re-growth (g)	Shoot FW after re-growth as % of initial shoot FW
**Batch 1**			n = 4	n = 6	
*L. peregrinus*	PI 368905	Annual	27.1±2.2	0	0
*L. ornithipodiodes*	PI 442514	Annual	33.7±1.8	0.2±0.1	0.7
*L. edulis*	PI 244281	Annual	38.8±1.5	0.3±0.2	0.7
*L. arabicus*	PI 214109	Annual	22.1±1.8	0.5±0.4	2.3
*L. japonicus* MG-20	n/a	Perennial	25.2±1.7	0.6±0.2	2.3
*L. subbiflorus*	PI 631785	Annual	25.9±1.6	1.4±0.3	5.3
*L. burttii*	n/a	Annual	15.8±0.3	1.3±0.2	8.1
*L. glinoides*	PI 246736	Annual	31.5±2.0	2.7±0.1	8.6
*L. japonicus* Gifu	n/a	Perennial	16.0±1.5	1.5±0.2	9.4
*L. uliginosus*	W6 20479	Perennial	28.7±2.1	3.8±0.5	13.2
*L. corniculatus*	PI 464684	Perennial	17.1±1.5	2.6±0.4	15.1
*L. tenuis* (*L. glaber*)	PI 302922	Perennial	19.0±0.8	2.9±0.4	15.3
*L. parviflorus*	PI 415815	Annual	28.6±1.8	4.5±0.6	15.8
**Batch 2**			n≥4	n = 6	
*L. denticulatus*	PI 236862	Annual	2.0±0.3	n.d.	n.d.
*L. unifoliolatus*	PI 338644	Perennial	11.4±0.6	0	0
*L. angustissimus*	PI 368894	Annual	7.0±1.2	0	0
*L. weilleri*	PI 196332	Annual	11.2±1.1	0.01±0.00	0.1
*L. halophilus*	PI 300237	Annual	12.6±0.3	0.03±0.03	0.3
*L. arenarius*	PI 319020	Annual	10.4±0.4	0.05±0.04	0.5
*L. conimbricensis*	PI 308033	Annual	7.5±0.5	0.08±0.03	1.1
*L. gebelia*	PI 464824	Perennial	5.4±0.5	0.08±0.03	1.5
*L. mearnsii*	PI 226275	Perennial	4.7±0.6	0.18 0.01	3.8
*L. japonicus* MG-20	n/a	Perennial	5.5±0.3	0.25±0.04	4.5
*L. japonicus* Gifu	n/a	Perennial	4.5±0.5	0.49±0.06	10.7
*L. collinus*	PI 641351	Annual	2.8±0.6	0.32±0.05	11.5
*L. palustris*	PI 311427	Perennial	3.4±0.3	0.62±0.09	18.42

Plants were grown in the glasshouse and analyses performed on four to five month old plants, as for Figure 2 and Figure 3. Plants in the first batch were grown in a glasshouse supplemented with artificial light (photoperiod of 16 h). Plants in the second were grown in a glasshouse under natural long days. Values are means ± SE of measurements on the number of plants indicated (n), 15 days after cutting back. n.d. – not determined; FW – fresh weight. Sources for the “USDA/GRIN and published life forms” are as follows: Germplasm Resource Information Network (GRIN, http://www.ars-grin.gov/) database; [9]; [10]; [11].

### Iodine Staining of Plant Tissues

Plant tissues were decolourised by heating in 80% (v/v) ethanol for 30 min at 70°C with further incubations in ethanol at RT over several hours as required for full decolourisation. Tissues were stained with iodine solution (Lugol’s solution) for 20 min then washed with water prior to imaging.

## Results

### Mutants of *Lotus japonicus* with Severely Reduced Starch Contents are Impaired in their Ability to Re-grow following Cutting Back

**Figure 1:**
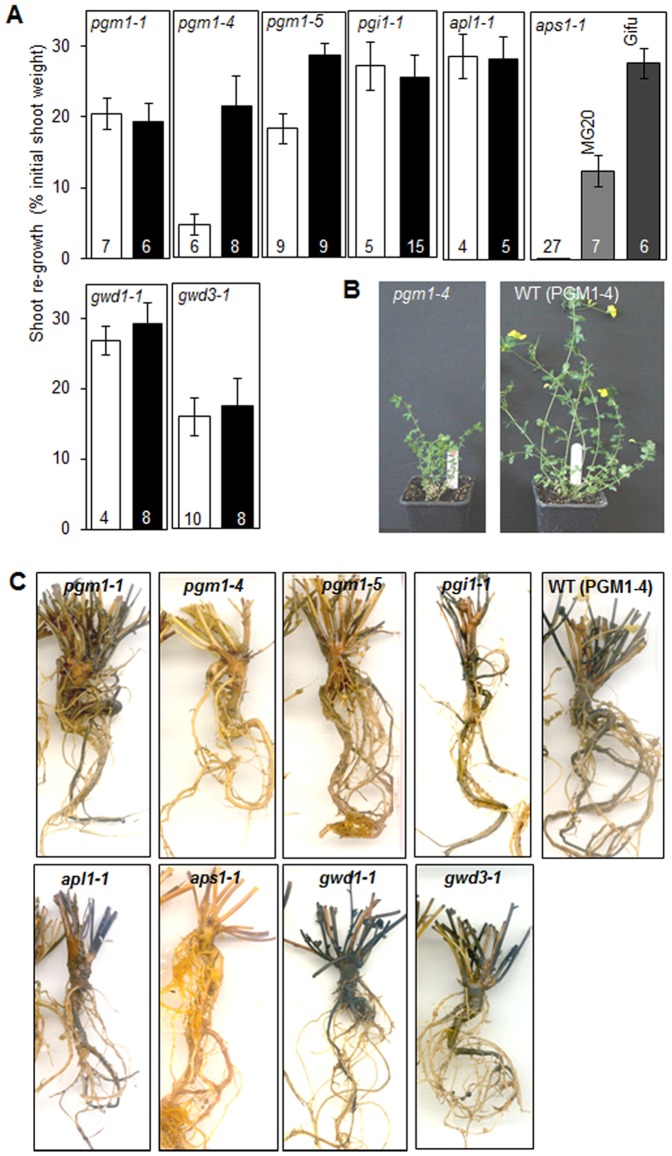
Effect of cutting back on growth of *L. japonicus* starch mutants. Plants of *pgm1-1*, *pgm1-4*, *pgm1-5*, *apl1-1, aps1-1*, *gwd1-1*, and *gwd1-3* and their wild-type (WT) segregants were grown in a glasshouse, supplemented with artificial light to provide long days. Plants of *pgi1-1, apl1-1* and their wild-types were grown in a glasshouse under natural long days. All mutants were cut back when four- to five-months old, except for *apl1-1* and its WT segregants which were 10 months old. (A) Shoot fresh weight six weeks after cutting, as a percentage of shoot fresh weight at the time of cutting. Each mutant (white bar) is compared with co-segregating wild-type plants from the same population (black bar), except for *aps1-1* which is compared with the parental wild-type lines Gifu and MG-20 as no segregants were available. Values are means from the number of biological replicates indicated on each bar, ± SE. Values differed significantly between mutant and wild-type plants (Student’s t test, p<0.05) for *pgm1-4*, *pgm1-5*, and *aps1-1* mutants but not for other mutants. (B) Appearance of *pgm1-4* and wild-type plants after re-growth. (C) Appearance of root systems of representative mutant plants and a representative wild-type plant (PGM1-4 segregant) following decolourisation and iodine staining (samples representative of at least three biological replicates). Note the lack of iodine staining in *pgm1-4* and *aps1-1* root systems.

To establish whether stored starch is necessary for re-growth capacity and vigour, we used a collection of *L. japonicus* starch metabolism mutants deficient in specific enzymes of either the starch synthesis or the starch degradation pathways as described in Materials and Methods. Whereas all of the mutants grew at approximately the same rate as wild-type plants in our glasshouse conditions, shoot re-growth after cutting back was seriously impaired relative to that of wild-type plants in three of the six starch synthesis mutants examined. These were the *pgm* mutant lines *pgm1-4* and *pgm1-5*, and the *aps1* mutant. For the *pgm* mutants, re-growth was reduced by ca. 40 to 80% relative to that of their wild-type segregants, and the *aps1* mutant showed no re-growth at all (Fig. 1A, B). Iodine staining showed that the roots, stem and shoot bases of all three genotypes contained little or no starch (Fig. 1C). Consistent with this observation, we showed previously that all three of these mutants have drastically reduced levels of starch throughout the plant [8].

All of the remaining starch synthesis mutants analysed – *pgm1-1*, *pgi1-1* and *apl1-1*– were not impaired in shoot re-growth relative to wild-type plants. These mutant lines had approximately the same amount of starch in roots, stems and stem bases as wild-type plants (Fig. 1), in agreement with our previous analyses in which we found these lines to have no severe reductions in root starch content [8]. Shoot re-growth was not affected in either of the two starch degradation mutants (*gwd1* and *gwd3*). Their root starch contents were similar to those of wild-type plants (Fig. 1).

### Root Starch Content and Re-growth Vigour in *Lotus* Species

The experiments above establish that starch stored in the roots is important for re-growth vigour in *Lotus japonicus*. To discover whether this finding is more widely applicable, we used a collection of both annual and perennial *Lotus* species containing wild and cultivated species from most of the major clades within the genus, and from different geoclimatic regions (Table 1, Fig. S1, S2).

Species of *Lotus* were analysed for their capacity to re-grow after cutting back, and for the vigour of re-growth. The collection was analysed in two batches; the *L. japonicus* accessions Gifu and MG-20 were represented in both batches. Differences in root starch content and re-growth between these two accessions were similar in the two batches demonstrating the datasets can be compared. For Gifu, re-growth was 9.4 and 10.7% of initial shoot weight in the first and second batches respectively, and for MG-20 these values were 2.3 and 4.5% in the first and second batches, respectively (Table 1).

**Figure 2:**
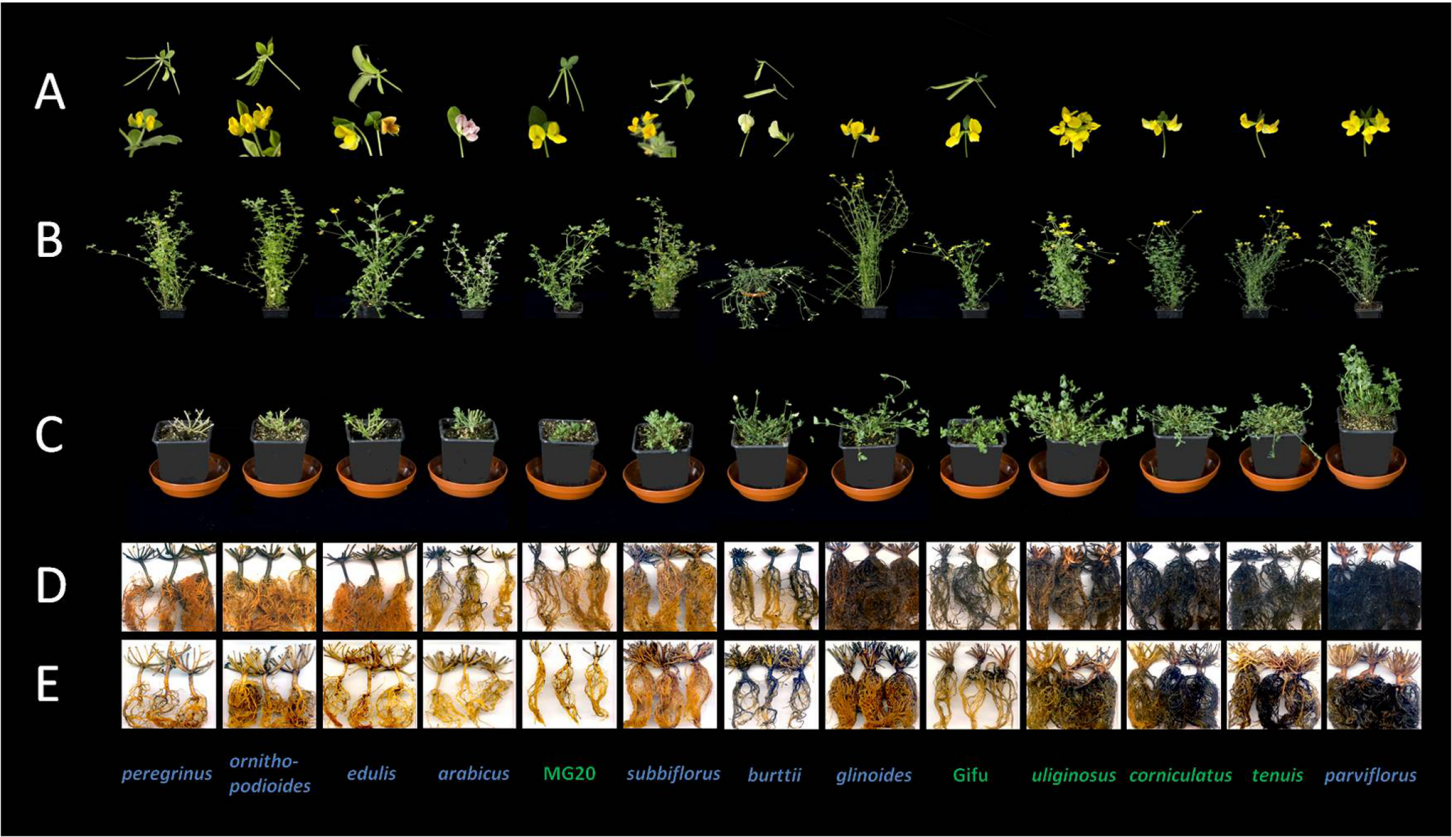
Effect of cutting back on *Lotus* species (batch 1). Plants were grown, harvested and iodine-stained as described in the legend of Table 1 and in Materials and Methods. (A) Flowers and seed pods at the time of cutting back. Where there is no picture the species had not flowered at the time of cutting back. Note that *L. arabicus, L. glinoides, L. uliginosus, L. corniculatus, L. tenuis* (aka *L. glaber*) and *L. parviflorus* had flowered, but not set seeds. (B) Plants at the point of cutting back. (C) Plants 15 days after cutting back. Plant species names are color-coded according to their life form: annual species in blue, perennial species in green (classification details given in Table 1). (D) and (E) Root systems of *Lotus* species following decolourisation and iodine staining before cutting back (D) and 15 days after cutting back (E).

**Figure 3:**
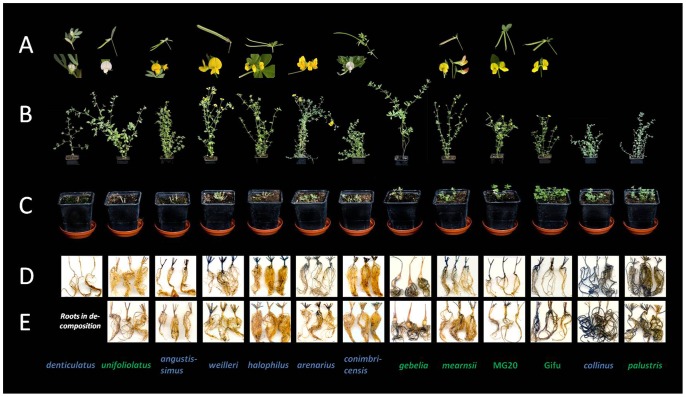
Effect of cutting back on *Lotus* species (batch 2). Plants were grown, harvested and stained as described in the legend of Table 1 and in Materials and Methods. (A) Flowers and seed pods at the time of cutting back. Where there is no picture the species had not flowered at the time of cutting back. Note that *L arenarius* had flowered, but not set seeds. (B) Plants at the point of cutting back. (C) Plants 15 days after cutting back. Plant species names are color-coded according to their life form: annual species in blue, perennial species in green (classification details given in Table 1). (D) and (E) Root systems of *Lotus* species following decolourisation and iodine staining before cutting back (D) and 15 days after cutting back (E).

All *Lotus* species, both annual and perennial, were able to re-grow at first (emergence of new shoots and leaves from stem buds ca. two days after cutting back). However, there were strong differences between genotypes in the vigour of re-growth. Whereas new growth on some genotypes died after a few days, other genotypes showed continuing growth with various degrees of vigour. There was a marked correlation between the extent of re-growth and the starch content in primary and secondary roots. All ten species with re-growth of greater than 8% of initial shoot weight had substantial root starch contents, whereas species in which re-growth was less than 5% generally had low or undetectable root starch (Fig. 2, 3). In contrast to the starch stored in primary and secondary roots, no clear correlation was seen between the starch stored in stems and shoot bases of the species and their re-growth vigour following cutting back. Starch was present in the stem and shoot base and was lost from these organs following cutting back in several species that lacked high re-growth capacity (for example *L. peregrinus*, *L. edulis* and *L. halophilus*). Conversely, starch content was relatively low in the stem and shoot base of *L. parviflorus*, one of the species with very vigorous re-growth (Fig. 2, 3).

In general, annual species of *Lotus* re-grew less vigorously than perennial species. In both batches, four out of the five most vigorous species were perennials and four out of five of the least vigorous were annuals according to the USDA/GRIN life form classification. There were some notable exceptions, especially *Lotus parviflorus*, which is classified as an annual, but has a large accumulation of root starch and a large capacity for re-growth (Table 1, Fig. 2, 3).

## Discussion

### Root Starch is Important for Re-growth after Cutting Back in *Lotus japonicus*

We showed previously that mutants lacking phosphoglucomutase and therefore devoid of starch are indistinguishable from wild-type plants throughout the life-cycle [8], suggesting that starch is not necessary for the normal growth of *L. japonicus* in glasshouse and controlled environments. Our work here, however, establishes unambiguously that a store of starch in roots is important for re-growth after cutting back.

When wild-type plants were cut back so that essentially all photosynthetic tissue was removed, re-growth from stem buds was rapid and vigorous. For four- to five-month old plants, about 20 to 30% of the original shoot mass was regained within six weeks. Equivalent re-growth was seen in mutant plants lacking starch in the leaves but not in the roots (plants lacking plastidial phosphoglucoisomerase), and mutants with mild reductions in starch content (e.g. plants carrying the *pgm1-1* allele of *PGM1*, and plants lacking the large subunit of AGPase). However mutants severely deficient in root starch – lacking either plastidial phosphoglucomutase or the small subunit of AGPase – showed either very limited re-growth or no re-growth under our experimental conditions. The severely impaired capacity of these mutants to re-grow after cutting back suggests that stored starch may be essential for survival in a natural environment in which plants may be defoliated by pathogen attack, grazing or harvest.

We suggest the requirement for root starch for re-growth comes from its use as a respiratory substrate to support root function following cutting back. Removal of photosynthetic tissue stops the supply of sugars from the shoot to the root. If starch is present in the roots, it can be immediately degraded to restore a supply of respiratory substrates, thus allowing continued growth and maintenance of root functions including water and nutrient uptake. These processes are essential for the subsequent re-growth of the shoot. In contrast, roots that lack starch will experience starvation following removal of photosynthetic tissue. The progressive, deleterious consequences for root growth and function are highly likely to compromise shoot re-growth.

Our suggestion that root starch is used as a respiratory substrate to support root function following cutting back is consistent with results from previous work on re-growth following cutting back in other perennial legumes. The starch content of the tap root of alfalfa fell by 70% in six days following cutting back. Re-growth had barely started at this point, and root starch loss was much greater than dry weight gain through new shoot growth [1]. In experiments in which intact alfalfa plants were labelled with ^13^C via supply of ^13^CO_2_ to leaves, then cut back, root respiration accounted for most of the loss of ^13^C from root starch, and up to 40% of starch reserves were used in respiration [7], [15].

### Root Starch Content is Correlated with both Re-growth Vigour and Life Form in Species of *Lotus*

Our study of re-growth vigour in species of *Lotus* is consistent with our observations on *L. japonicus* mutants. Initial growth vigour, prior to cutting back, was independent of root starch content. However following cutting back there was an obvious correlation across species between the vigour of re-growth and the starch content of the roots.

Examination of this range of *Lotus* species also revealed that the roots of most perennial species stored significant amounts of starch, whereas the roots of annual species of the same age and growth stage generally contained little or no starch. These results are in agreement with the general view that photosynthates tend to be allocated largely to reproduction in annuals, but less to reproduction and more to vegetative storage in perennials (as in, for example, studies of annual and perennial species of *Gossypium*; [16], [17]). In general, perennial species with significant root starch contents re-grew better than annual species with little or almost no root starch.

There were a few notable exceptions to the general correlation between the annual habit, lack of root starch, and poor re-growth. *L. parviflorus* and *L. collinus* had high root starch contents and vigorous re-growth, but are classified as annuals. While a range of factors in addition to possession of root starch is likely to be important in determining the perennial habit (see below), we suggest that the life-form of these two species should be re-examined in a range of environmental conditions. It may be that they behave as perennials under some conditions. It is also worth noting that some of *Lotus* species analysed in our study did not produce seeds under our growth conditions. This might have influenced the allocation of carbon to the roots.

Altogether, our work establishes that a store of starch in the roots is important for re-growth after cutting back in perennial legume species such as *L. japonicus.* The correlation between root starch levels, the capacity for re-growth, and the life form of the species suggests that root starch storage supports important features of the perennial habit. First, mobilisation of root starch may enhance the supply of carbon for the production of new vegetative growth following flowering. Second, the long-term survival of herbaceous perennials like these *Lotus* species may depend on their capacity for vigorous re-growth following loss of photosynthetic capacity, for example to grazing or during winter. However, although starch storage probably confers advantages on perennial plants it seems unlikely that it is a primary determinant of the perennial habit. Recent research on species of Arabidopsis suggests that perenniality is closely tied to patterns of expression of transcription factors that mediate the transition of apical meristems from an indeterminate vegetative state to a determinate, reproductive state. These transcription factors – including FLOWERING LOCUS C (FLC) and SUPPRESSOR OF OVEREXPRESSION OF CONSTANS 1 (SOC1) – also directly and indirectly influence a range of other developmental, metabolic and stimulus-perception processes within the plant (e.g. [18]–[21]). It seems likely that they coordinate programmes of gene expression that confer advantages for either annual or perennial growth. We speculate that vegetative carbohydrate storage may be linked in this way with the perennial habit.

Of more than 180 *Lotus* species, only four have been domesticated and improved by plant breeding - *L. corniculatus, L. tenuis* (also named *L. glaber*), *L. subbiflorus* and *L. uliginosus* and are of agricultural importance, especially in South America [22]. Our findings suggest that *L. parviflorus* (USDA accession PI 415815) may also have agronomic potential. This species out-performed all the others studied here, both for the amount of starch stored in its roots and its re-growth vigour following cutting back. In addition, *L. parviflorus* had the largest root biomass of all the species analysed. These characteristics are interesting for further studies of starch metabolism and re-growth vigour, and may also be valuable for attempts to breed improved *Lotus* forage crops, especially where persistence or biomass yield is a target trait [12], [22].

## Supporting Information

Figure S1 **Phylogenetic relationship of the species of the genus** ***Lotus*** **and their life forms.** Modified from [12]. *Lotus* species that were analysed in this study are framed. Annual species are framed in blue, perennial in green (USDA GRIN/published life form). Species included in the collection used in this study but not included in the phylogenetic analysis of [12] are mentioned on the right bottom of the Figure. More detailed phylogenetic analyses including the position of *L. burttii* in the tree can be found in [13]. Note that the annual and perennial species are largely intermixed, suggesting that the switch of one life form to another may not require major genetic changes.
(PDF)

Figure S2 **Root starch content, re-growth vigour, life form, and geoclimatic origin of the** ***Lotus*** **species used in this study.** Geoclimatic origin (geographic origin as given by the USDA GRIN ‘Taxonomy for Plants’ database and the website http://www.globalspecies.org, and geoclimatic information as given by the ‘World map of the Köppen-Geiger climate classification’ published in [14], USDA GRIN/published life form, root starch content, and re-growth vigour following cutting of the the annual and perennial species of *Lotus* analysed in the cutting-back experiment. Scores were attributed to the *Lotus* natural variants for their root starch content and re-growth vigour based on results of biomass measurements and root iodine staining as described in main text and in the legend of Table 1, Figure 2, 3.
(PDF)
