# Supplementary figures and images for: Root Starch Reserves Are Necessary for Vigorous Re-Growth following Cutting Back in Lotus japonicus
Source: PLoS One. 2014 Jan 31;9(1):e87333. doi: 10.1371/journal.pone.0087333 (PMC3909078; doi:10.1371/journal.pone.0087333)

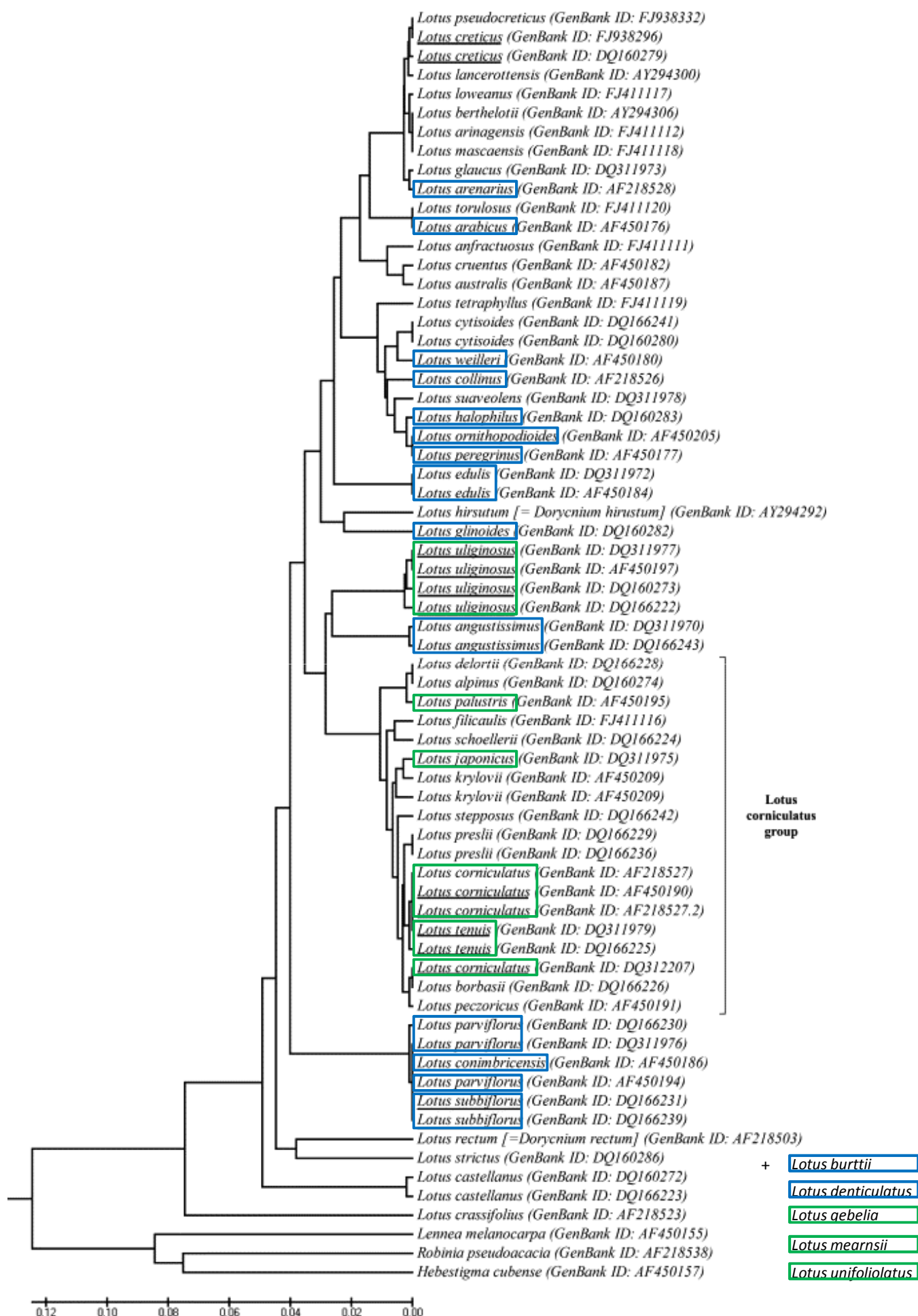

Supplement: Figure S1 — Phylogenetic relationship of the species of the genus Lotus and their life forms. Modified from [12]. Lotus species that were analysed in this study are framed. Annual species are framed in blue, perennial in green (USDA GRIN/published life form). Species included in the collection used in this study but not included in the phylogenetic analysis of [12] are mentioned on the right bottom of the Figure. More detailed phylogenetic analyses including the position of L. burttii in the tree can be found in [13]. Note that the annual and perennial species are largely intermixed, suggesting that the switch of one life form to another may not require major genetic changes. (PDF) [file pone.0087333.s001.pdf]
