# Supplementary material for: Root Starch Reserves Are Necessary for Vigorous Re-Growth following Cutting Back in Lotus japonicus
Source: PLoS One. 2014 Jan 31;9(1):e87333. doi: 10.1371/journal.pone.0087333 (PMC3909078; doi:10.1371/journal.pone.0087333)
Supplement: Figure S2 — Root starch content, re-growth vigour, life form, and geoclimatic origin of the Lotus species used in this study. Geoclimatic origin (geographic origin as given by the USDA GRIN ‘Taxonomy for Plants’ database and the website http://www.globalspecies.org, and geoclimatic information as given by the ‘World map of the Köppen-Geiger climate classification’ published in [14], USDA GRIN/published life form, root starch content, and re-growth vigour following cutting of the the annual and perennial species of Lotus analysed in the cutting-back experiment. Scores were attributed to the Lotus natural variants for their root starch content and re-growth vigour based on results of biomass measurements and root iodine staining as described in main text and in the legend of Table 1, Figure 2, 3. (PDF) [file pone.0087333.s002.pdf]

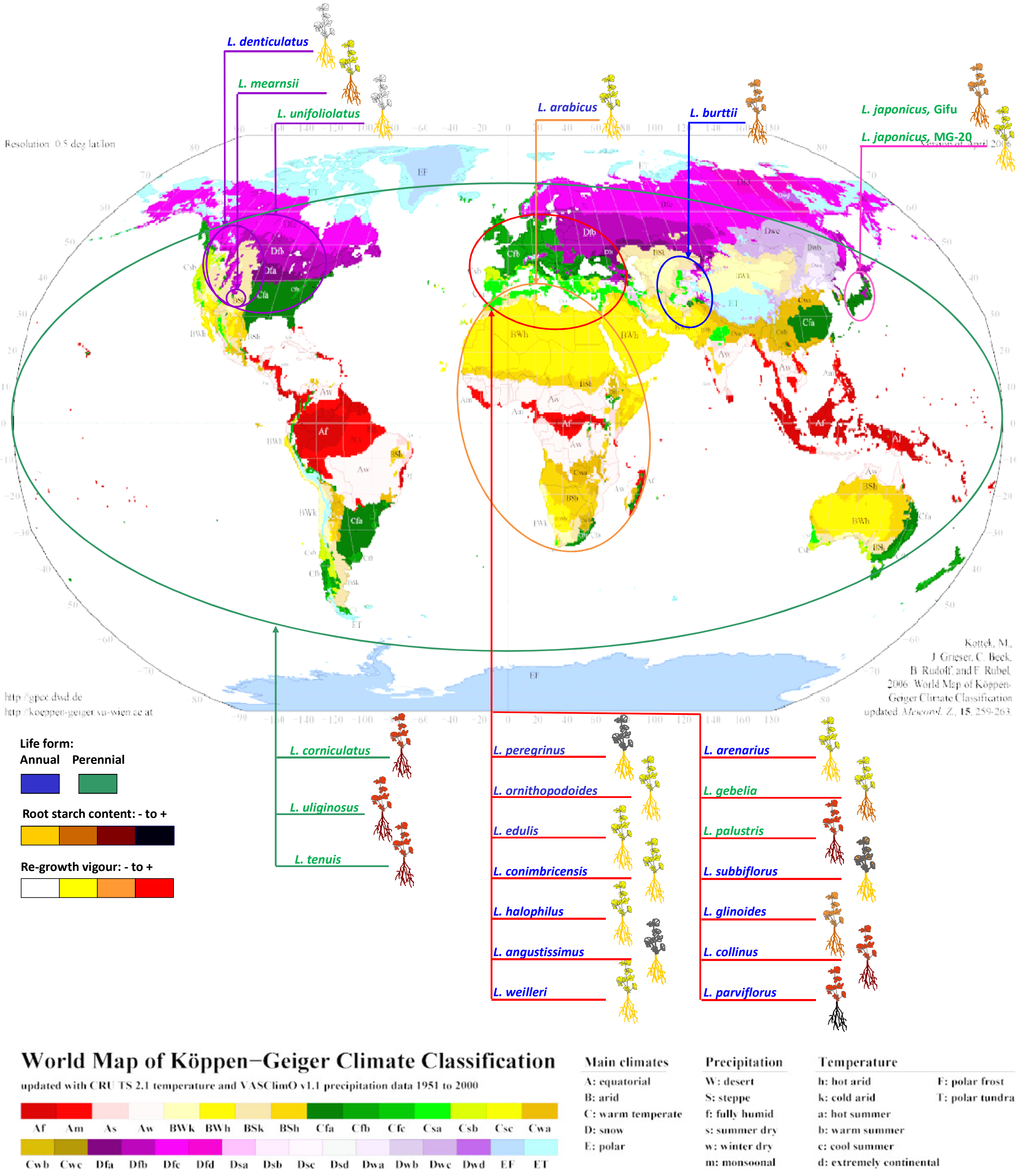

L. denticulatus
L. mearnsii
L. unifoliolatus
L. arabicus
L. burttii
L. japonicus, Gifu
L. japonicus, MG-20
Resolution 0.5 deg lat/lon
Version of April 2006
Kottek, M., J. Grieser, C. Beck, B. Rudolf, and F. Rubel, 2006: World Map of Köppen-Geiger Climate Classification updated. Meteorol. Z., 15, 259-263.
http://gpcc.dwd.de
http://koeppen-geiger.vu-wien.ac.at
Life form:
Annual
Perennial
Root starch content: - to +
Re-growth vigour: - to +
L. corniculatus
L. uliginosus
L. tenuis
L. peregrinus
L. ornithopodioides
L. edulis
L. conimbricensis
L. halophilus
L. angustissimus
L. weilleri
L. arenarius
L. gebelia
L. palustris
L. subbiflorus
L. glinoides
L. collinus
L. parviflorus
World Map of Köppen−Geiger Climate Classification
updated with CRU TS 2.1 temperature and VASClimO v1.1 precipitation data 1951 to 2000
Af Am As Aw BWk BWh BSk BSh Cfa Cfb Cfc Csa Csb Csc Cwa
Cwb Cwc Dfa Dfb Dfc Dfd Dsa Dsb Dsc Dsd Dwa Dwb Dwc Dwd EF ET
Main climates
A: equatorial
B: arid
C: warm temperate
D: snow
E: polar
Precipitation
W: desert
S: steppe
f: fully humid
s: summer dry
w: winter dry
m: monsoonal
Temperature
h: hot arid
k: cold arid
a: hot summer
b: warm summer
c: cool summer
d: extremely continental
F: polar frost
T: polar tundra
